# Friction Behavior of a Textured Surface against Several Materials under Dry and Lubricated Conditions

**DOI:** 10.3390/ma14185228

**Published:** 2021-09-11

**Authors:** Linqing Bai, Jianxin Sun, Pengcheng Zhang, Zulfiqar Ahmad Khan

**Affiliations:** 1 School of Mechanical and Automotive Engineering, Qingdao University of Technology, Qingdao 266520, China; snjnxn@163.com (J.S.); zhangpengcheng6538@163.com (P.Z.); 2 NanoCorr, Energy & Modelling (NCEM) Research Group, Department of Design and Engineering, Bournemouth University, Dorset BH12 5BB, UK; ZKhan@bournemouth.ac.uk

**Keywords:** surface texture, friction behavior, wear, dry friction, lubricated condition

## Abstract

This paper reports research on the frictional behavior of a textured surface against several materials under dry and lubricated conditions, and this is aimed to provide design guidelines on the surface texturing for wide-ranging industrial applications. Experiments were performed on a tribo-tester with the facility of simulating A ball-on-plate model in reciprocating motion under dry, oil-lubricated, and water-lubricated conditions. To study the frictional behavior of textured SiC against various materials, three types of ball-bearing –elements, 52100 steel, silicon nitride (Si_3_N_4_), and polytetrafluoroethylene (PTFE), were used. Friction and wear performance of an un-textured surface and two types of widely used micro-scale texture surfaces, grooves and circular dimples, were examined and compared. The results demonstrated that the effect of surface textures on friction and wear performance is influenced by texture parameters and the materials of friction pairs. The circular-dimple texture and the groove texture, with certain texture parameters, played a positive role in improving friction and wear performance under specific operating conditions used in this research for SiC–steel and SiC–Si_3_N_4_ friction pairs; however, there was no friction and wear improvement for the textured SiC–PTFE friction pair. The results of this study offer an understanding and a knowledge base to enhance the performance of bearing elements in complex interacting systems.

## 1. Introduction

For equipment which works under extreme conditions under extreme conditions, a poor lubrication state of the key parts severely restricts the service performance of the whole machine or system. For example, the contact wear of rough peaks on the friction pair surface caused by heavy load or by insufficient oil supply will affect the reliability and service life of the equipment. How to effectively reduce equipment failure caused by severe friction and wear under extreme conditions has become a common issue that needs to be resolved.

In the past decades, as surface processing technology has developed, surface texture has become a subject of attention within the interacting systems and complex machine elements to reduce friction and wear, such as mechanical interacting components, elements, piston liners, and rings and cutting tools [1,2,3,4,5]. Surface texturing modifies surface topography by constructing micro- and/or nano-scale dimples or grooves on a machined surface by micro-/nanofabrication processes, such as laser ablation, photochemical machining, electric discharge machining, and photochemical machining [6,7,8,9,10]. The use of surface textures has impacts on several key attributes that include the actual area of contact, the type of lubricating fluid, contact pressure, and the distribution of wear debris in the contact zone. Under various lubrication conditions, the effects of surface texture on frictional performance and tribological wear are varied. Under a dry-friction condition, the surface texture mainly plays a role in storing debris and preventing further severe wear [11]. However, under a lubricated condition, the mechanism of surface texturing is more diversified, which can play a role in not only increasing hydrodynamic pressure and lubricating oil storage but also grinding debris capture [12].

Previously, many studies have reported the effects of a textured surface on frictional performance. Etsion et al. [1] researched the frictional performance of textured seal surfaces. An optimum-size dimple, which can reduce the friction torque by about 50%, was evidenced. Wang et al. [13] found that optimum micro-pits can increase the load-carrying capacity of SiC bearings up to about 2.5 times over that of an un-textured surface. The research of Dizdar et al. [14] and Ajayi et al. [15] demonstrated that the geometrical orientation of textures significantly influences lubrication performance. When the texture direction was right-angled to the direction of sliding, the friction could be improved by a reasonable design of other geometric parameters of the textures. However, when the texture orientation was in line with the direction of motion, the friction and wear increased.

Most previous studies on surface texturing have focused on optimizing texture parameters for optimal frictional performance, such as the texture size, shape, depth, and arrangement [16,17,18,19,20,21,22,23]. However, there is a significant gap in terms of studies that deal with the influence of friction pair materials on the frictional characteristics of textured surfaces, which limits the application of surface textures. The main objective of this work is to research the influence of friction pair materials on the wear and friction of interacting surfaces with artificially induced or designed textured configurations subjected to various lubrication conditions. During testing, the frictional coefficients of two broadly used features, circular dimples and grooves, were measured under dry, oil-lubricated, and water-lubricated conditions with the three friction pairs of SiC–steel, SiC–Si3N4, and SiC–PTFE. After friction testing, the wear performance was also analyzed. This research demonstrated positive effects and also provided valuable insights into the applications of surface textures with several types of friction pair materials.

## 2. Methods and Materials

### 2.1. Experimental Device

**Figure 1:**
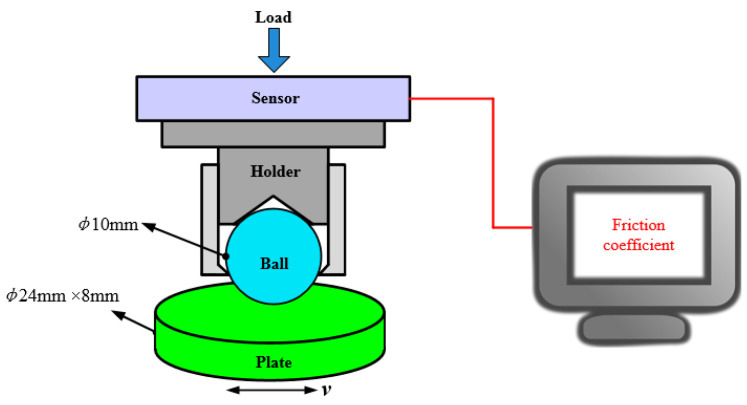
Schematic of the test rig.

The frictional behavior of a textured silicon carbide (SiC) plate against several types of materials was tested on an experimental device named UMT-2 (Center for Tribology, Inc. Campbell, CA, USA) with a ball-on-plate modular simulator (Center for Tribology, Inc. Campbell, CA, USA), as depicted in Figure 1. The stationary upper ball was fixed on a holder, and the lower plate moved reciprocally with a set frequency. The operating conditions used in this research are shown in Table 1.

### 2.2. Samples and Lubricants

#### 2.2.1. Samples

To investigate the frictional behavior of textured SiC against various types of materials, three types of ball-bearing elements of materials 52100 steel, Si_3_N_4_, and PTFE were used in the experiments, which were named Ball-1, Ball-2, and Ball-3. Table 2 shows the properties of the plate and ball samples.

**Figure 2:**
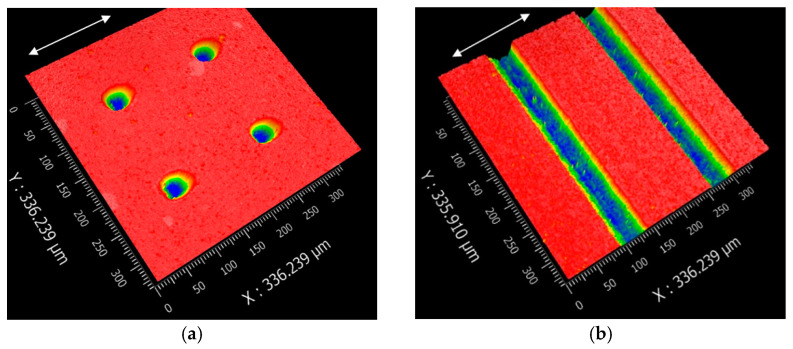

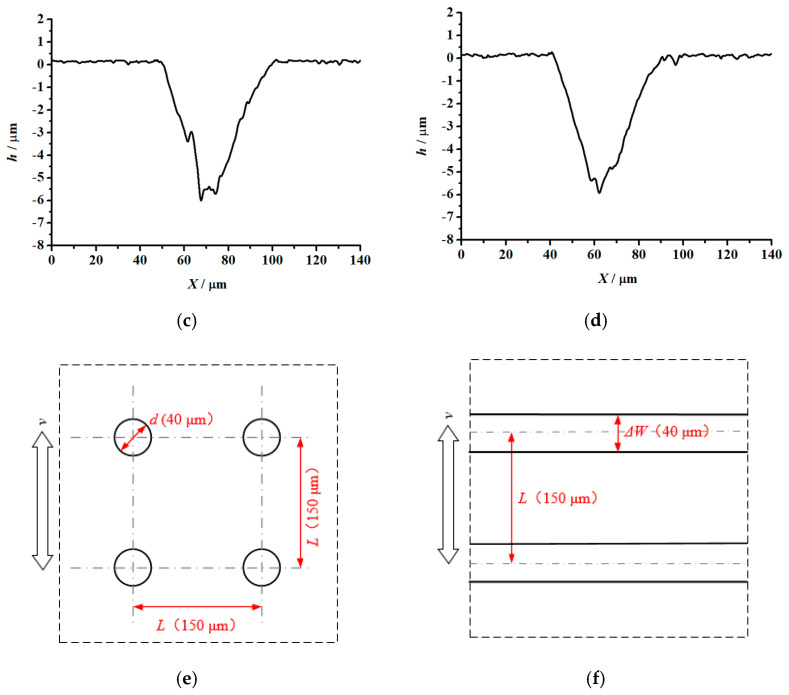
3-D and 2-D profiles of the textured SiC plate surface: (**a**) 3-D profile of the circular-dimple-textured surface with a depth of 5 μm, (**b**) 3-D profile of the groove-textured surface with a depth of 5 μm, (**c**) 2-D profile of (**a**), (**d**) 2-D profile of (**b**), (**e**) distribution diagram of circular dimples, and (**f**) distribution diagram of grooves.

To compare the frictional performance of the textured and un-textured SiC plates, picosecond laser with a wavelength of 532 nm and a pulse frequency of 100 kHZ was used to fabricate textures on the plate surfaces. In the texture-machining process, 0.3 W laser energy and 100 mm/s scanning speed were selected to ensure that processing quality and efficiency were sustained. After laser processing, no further processing was performed on the surface. Figure 2 shows the 3-D and 2-D profiles of circular-dimple-textured and groove-textured plate surfaces. Dimples and grooves had the same depth and center distance. Since it is reported that texture depth influences friction performance significantly, three different depths of 3, 5, and 10 μm were selected. The center distance was fixed at 150 μm for all textures. In this study, the dimple diameter *d* and the groove width Δ*W* were set to about 50 μm. The white arrows in Figure 2 show the sliding directions of the textured samples. For comparison, the un-textured plate samples were also tested for reference.

#### 2.2.2. Lubricants

To study the frictional performance of the textured SiC plate under dry, water-lubricated, and oil-lubricated conditions, distilled water and synthetic base oil PAO4 were used as lubricants. The parameters of PAO4 are shown in Table 3.

### 2.3. Experimental Procedure

**Figure 3:**
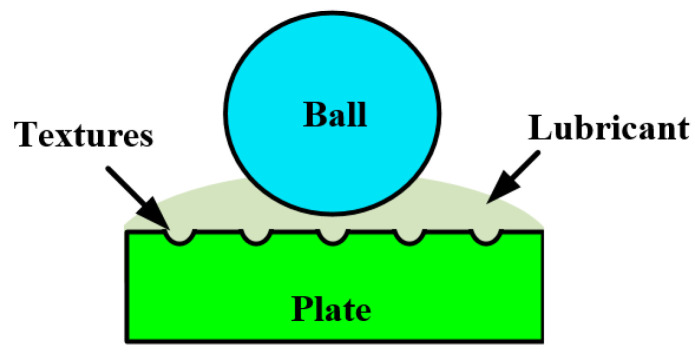
Schematic diagram of lubrication conditions.

Before the experiments, the samples were cleaned in petroleum and then acetone and alcohol. After that, they were dried with compressed nitrogen. Under the lubrication condition, 500 μL of distilled water/PAO4 was dripped on the plate before the test to ensure sufficient lubricant supply. In contrast, under the dry-friction condition, no lubricant was supplied. Figure 3 shows the schematic of the lubrication condition. The testing programs are shown in Table 4. Under the given testing conditions, the contact pressure and contact radius were calculated [24] and are shown in Table 5. At this contact pressure, the friction pair can be guaranteed to slide under harsh contact conditions.

## 3. Experimental Results

### 3.1. Dry Friction

**Figure 4:**
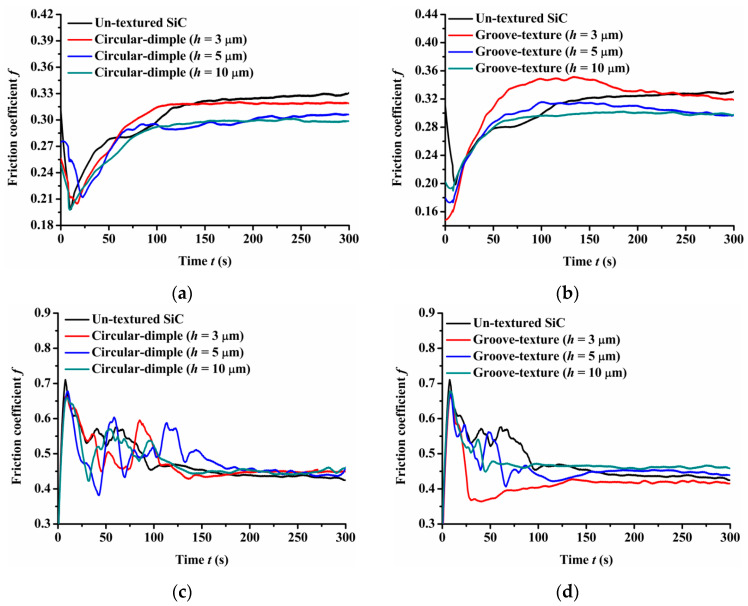

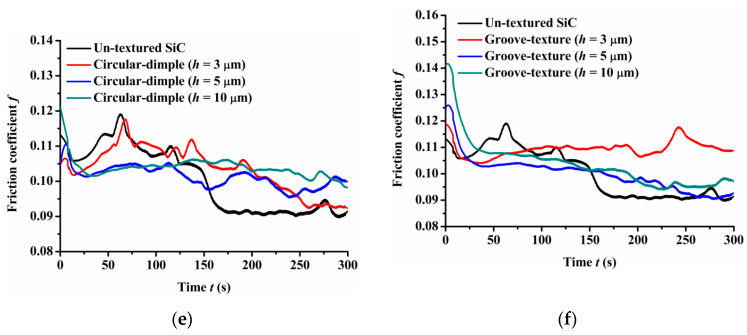
Friction performance of textured SiC under dry-friction conditions with a load of 30 N: (**a**) circular-dimple-textured SiC–steel ball, (**b**) groove-textured SiC–steel ball, (**c**) circular-dimple-textured SiC–Si_3_N_4_ ball, (**d**) groove-textured SiC–Si_3_N_4_ ball, (**e**) circular-dimple-textured SiC–PTFE ball, and (**f**) groove-textured SiC–PTFE ball.

Figure 4 provides the experimental friction performance of the un-textured, circular-dimple-textured, and groove-textured SiC surfaces with various texture depths under dry-friction conditions. The coefficient of friction of the three types of friction pairs, SiC–steel friction pair, SiC–Si_3_N_4_ friction pair, and SiC–PTFE friction pair, were tested.

As shown in Figure 4a, when a steel ball was used, the circular-dimple-textured surface exhibited a lower friction coefficient value than the un-textured surface. As the dimple depth increased from 3 μm to 10 μm, the friction coefficient decreased. For the groove-textured plate, as shown in Figure 4b, the friction coefficient also decreased as the texture depth increased. However, when the groove depth was 3 μm, the friction coefficient of the textured surface was greater than that of the un-textured surface for most of the friction experimental time, although the final friction coefficient was still less than that of the non-textured surface. This may be because in the dry-friction state, it is easy to produce grinding. Both circular-dimple and groove textures had the ability to accommodate the grinding to reduce abrasive particle wear, so the friction coefficient was lower relative to the un-textured surface. However, for the groove-textured surface, the texture area was relatively larger than that of the circular-dimple-textured surface; therefore, the contact pressure was larger, causing more abrasive wear. For the groove texture with a depth of 3 μm, the texture was not deep enough to accommodate the grinding, leading to a larger friction coefficient than that of the non-textured surface.

When a Si_3_N_4_ ball was used, the friction coefficient showed different trends compared to the steel ball, as seen in Figure 4c,d. It was lower for the circular-dimple-textured surface in the initial 100 s and then reduced to a value near to or larger than that of the un-textured surface (Figure 4c). For the grooved surface having various nadirs, the friction coefficient increased as the groove depth increased. When the depth was 3 μm, the friction coefficient was lower than that of the un-textured surface. However, when the depth increased to 5 and 10 μm, the friction coefficient was larger than of the un-textured surface after about 150 s sliding.

When the friction pair was SiC–PTFE, the friction coefficient of the circular-dimple-textured and groove-textured surfaces was larger than that of the un-textured surface finally. This means that when the ball was PTFE, the circular-dimple and groove textures with various depths showed no improvement in frictional performance.

Comparing the friction pairs of SiC–steel, SiC–Si_3_N_4_, and SiC–PTFE, the friction behavior showed significantly dissimilar trends for textured SiC against various types of materials under dry conditions. The SiC–steel friction pair significantly reduced the friction coefficient when textured circular-dimple and groove textures were fabricated on the SiC plate. When the SiC–Si_3_N_4_ friction pair was used, only groove textures with a depth of 3 μm exhibited better performance. When the PTFE ball was used, the circular-dimple and groove textures with various depths showed absolutely no friction improvement at all.

### 3.2. Oil-Lubricated Condition

**Figure 5:**
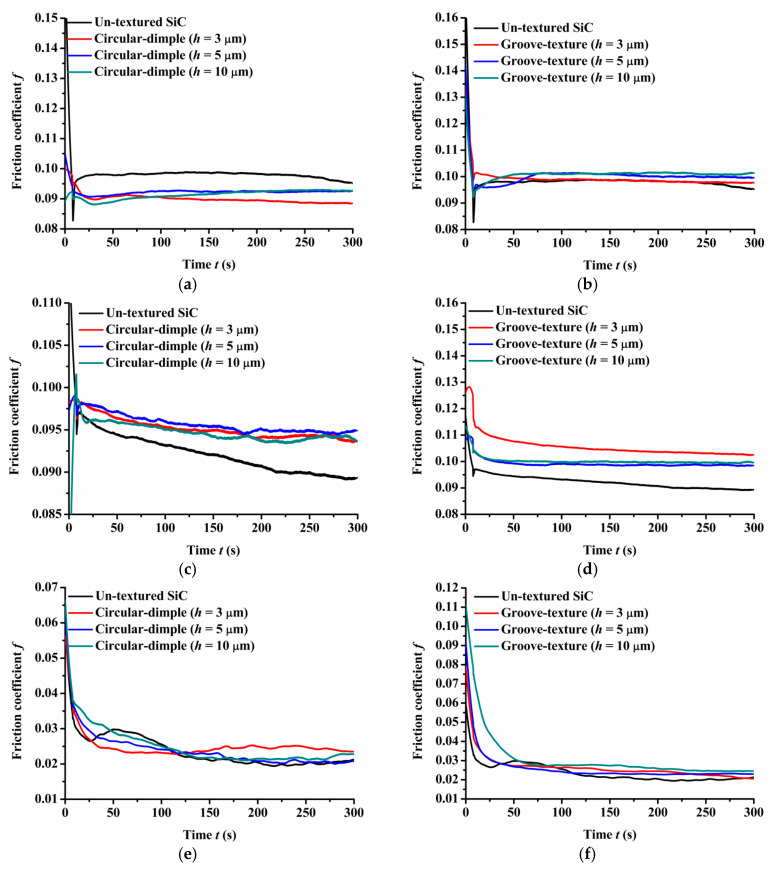
Friction performance of textured SiC under oil-lubricated conditions with a load of 75 N: (**a**) circular-dimple-textured SiC–steel ball; (**b**) groove-textured SiC–steel ball; (**c**) circular-dimple-textured SiC–Si_3_N_4_ ball; (**d**) groove-textured SiC–Si_3_N_4_ ball; (**e**) circular-dimple-textured SiC–PTFE ball; (**f**) groove-textured SiC–PTFE ball.

Figure 5 shows the experimental friction performance of the un-textured, circular-dimple-textured, and groove-textured SiC surfaces with various depths under oil-lubricated conditions. For the circular-dimple-textured plate samples with various depths, the friction coefficient was lower than that of the un-textured samples when the steel ball was used, as shown in Figure 5a. For all the groove-textured plates, as shown in Figure 5b, the friction coefficient remained almost constant but was greater than that of the un-textured surface throughout the experiments. The difference in the friction coefficient between the circular-dimple-textured and groove-textured samples was induced by the effect of a combination of contact pressure and hydrodynamic pressure produced by the textures. A larger contact pressure of the groove-textured surface caused a larger friction coefficient.

When the Si_3_N_4_ ball was used, the friction coefficient values showed different trends than those with the steel ball, as shown in Figure 5c,d. The value was much larger for both circular-dimple-textured surfaces and groove-textured surfaces with various depths than that for the un-textured surface. This means that when the friction pair was SiC–Si_3_N_4_, textures exacerbated the wear of the friction pair, so the friction coefficient increased.

When the friction pair was SiC–PTFE, the friction coefficient of circular-dimple-textured and groove-textured surfaces was slightly larger than that of the un-textured surface, but the difference in the coefficient values was not large. This means that when the ball was PTFE, the circular-dimple and groove textures with various depths showed no improvement in frictional performance.

By comparing the friction pairs of SiC–steel, SiC–Si_3_N_4_, and SiC–PTFE, it can be seen that the friction behavior showed quite different trends when textured SiC was used against several types of materials under oil-lubricated conditions. The SiC–steel friction pairs can significantly reduce the friction coefficient when a circular-dimple texture was fabricated on the SiC plate. However, the groove texture had no improvement as the circular-dimple texture. When the SiC–Si_3_N_4_ friction pair was used, none of the circular-dimple and groove textures showed better performance and even increased friction. When the PTFE ball was used, the circular-dimple and groove textures with various depths showed absolutely no friction improvement at all.

### 3.3. Water-Lubricated Condition

Silicon carbide ceramic shows an extremely low friction coefficient under water-lubricated simulated conditions. However, the viscosity of water is lower as compared with oil, leading to a smaller load capacity and the surface is susceptible to wear. Surface textures have evidently demonstrated to be an efficient system for improving tribological properties. To further improve the tribological performance of SiC ceramics, this study presents the friction coefficient of textured SiC combined with three types of mating materials under water-lubricated conditions.

**Figure 6:**
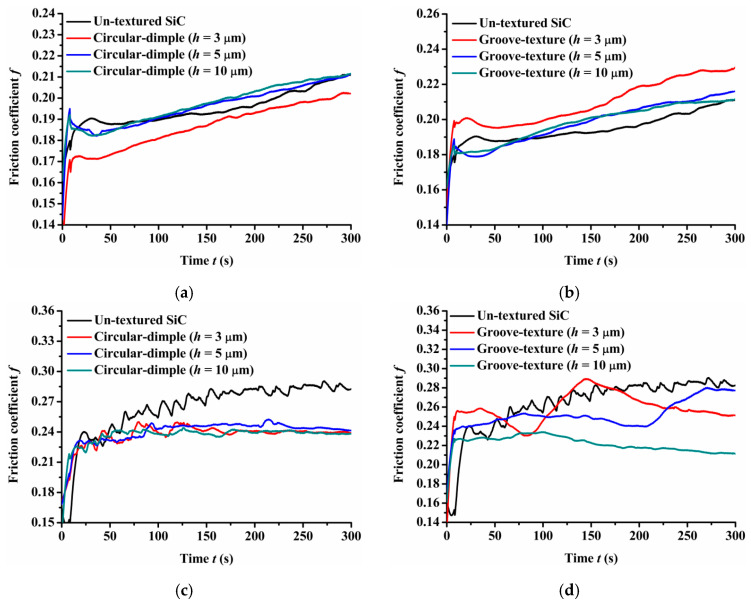

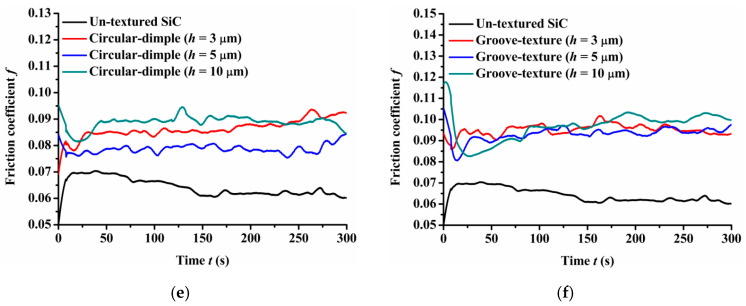
Friction performance of textured SiC under water-lubricated conditions with load of 75 N: (**a**) circular-dimple-textured SiC–steel ball, (**b**) groove-textured SiC–steel ball, (**c**) circular-dimple-textured SiC–Si_3_N_4_ ball, (**d**) groove-textured SiC–Si_3_N_4_ ball, (**e**) circular-dimple-textured SiC–PTFE ball, and (**f**) groove-textured SiC–PTFE ball.

Figure 6 shows that the experimental friction performance of SiC plates under water-lubricated conditions. As shown in Figure 6a, when a steel ball was used, the circular-dimple-textured surfaces had a lower friction coefficient compared to the un-textured surface when the dimple depth was 3 μm. As the dimple depth increased to 5 and 10 μm, the friction coefficient increased and was larger than that of the un-textured surface. For groove-textured plates as shown in Figure 6b, all friction coefficients were higher than that of the un-textured surface when the groove depths were 3, 5, and 10 μm. This means that under water lubrication conditions, the influence of textures on friction performance is mainly caused by hydrodynamic effect when the friction pair is SiC and steel. When the textured depth was more or the textured area was large, the hydrodynamic effect was weakened, leading to increased friction coefficient.

When the friction pair was SiC-PTFE, the friction coefficient trend was different to SiC-steel friction pair, as shown in Figure 6c,d. The friction coefficient of circular bumpy surface has been lesser than that of the un-textured surface as presented in Figure 6c. Under this condition, the texture depth had little influence on the friction coefficient. Whereas, for the groove-textured surface, the friction coefficient was also lower than that of the un-textured surface similar to the circular-dimple-textured surface. The groove depth had a stronger effect on the friction performance than the circular-dimple-textures as the values of friction coefficient had relatively large dispersibility. When the depth was 3 μm, the friction coefficient fluctuations were relatively large, but the value was lower than that of the un-textured surface for most of the sliding time.

When the material of the ball was PTFE, the friction coefficient of the circular-dimple-textured and groove-textured surfaces were much larger than that of the un-textured surface, as is shown in Figure 6e,f. This means that when the ball was PTFE, the circular-dimple and groove textures with various depths had no improvement in frictional performance.

Comparing the friction pairs of SiC–steel, SiC–Si_3_N_4_, and SiC–PTFE, the friction behavior showed quite different trends when the textured SiC against several types of materials under water-lubricated conditions. The SiC–Si_3_N_4_ friction pairs can significantly reduce friction coefficient when the circular-dimple and groove textures were fabricated on the SiC plate. Whereas, when the SiC–steel friction pair was used, only circular-dimple textures with a depth of 3 μm showed better performance. The friction performance was deteriorated when the textured circular dimples and grooves were used as the friction pair was SiC–PTFE.

## 4. Discussion

Experimental results are presented in Section 3. The friction performance of the textured surfaces evidenced varying trends subject to dry and lubricated conditions. The key function attributes of the surface texture within the context of tribological design are categorized into three distinct aspects. These aspects say (i) the surface texture can enhance the fluid hydrodynamic effect and in turn produce additional bearing capacity, (ii) textures have the capacity of storing a lubricant and of providing the “secondary supply” of lubrication to the contact area when and if the lubricant is scarce between the interacting surfaces within the maximum pressure contact area, and (iii) textures have the ability to reduce abrasive particles and the related wear by accommodating abrasive grains as refugee pockets. The effects of surface texture on friction and wear performance vary subject to various lubrication conditions. The friction behavior of a textured SiC surface at the interface of several materials, under both dry and lubricated conditions, were studied. The above three categories have significant impacts on the friction performance of the textured surface subject to lubricated conditions. The third category plays an important role in terms of friction performance under dry-friction conditions. It is worth noting that in addition to the impacts as mentioned above, surface texture can also contribute to friction performance. Contact pressure has an influence over friction performance, which has been demonstrated by research shown earlier. The presence of texture makes the contact area geometrically decrease, which in turn increases contact pressure, resulting in increased chance of wear. Therefore, the impact of texture on friction and wear performance is a cumulative result of the three above-mentioned aspects.

**Figure 7:**
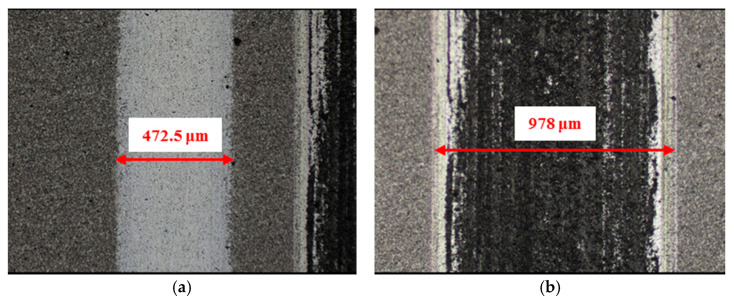
Wear performance of un-textured SiC under dry-friction conditions with a load of 30 N (×10): (**a**) un-textured SiC–steel ball and (**b**) un-textured SiC–Si_3_N_4_ ball.

**Figure 8:**
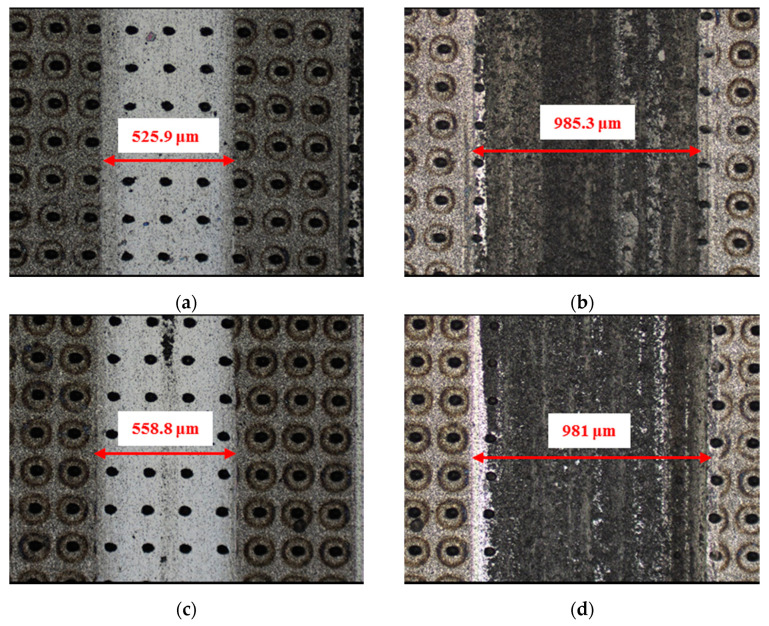

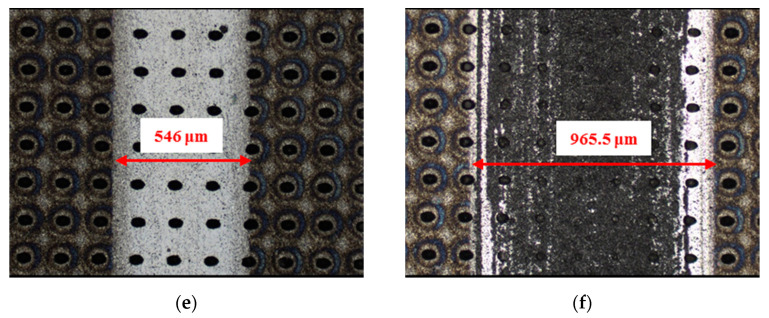
Wear performance of circular-dimple-textured SiC under dry-friction conditions with a load of 30 N (×10): (**a**) 3 μm circular-dimple-textured SiC-steel ball, (**b**) 3 μm circular-dimple-textured SiC–Si_3_N_4_ ball, (**c**) 5 μm circular-dimple-textured SiC–steel ball, (**d**) 5 μm circular-dimple-textured SiC–Si_3_N_4_ ball, (**e**) 10 μm circular-dimple-textured SiC–steel ball, and (**f**) 10 μm circular-dimple-textured SiC–Si_3_N_4_ ball.

**Figure 9:**
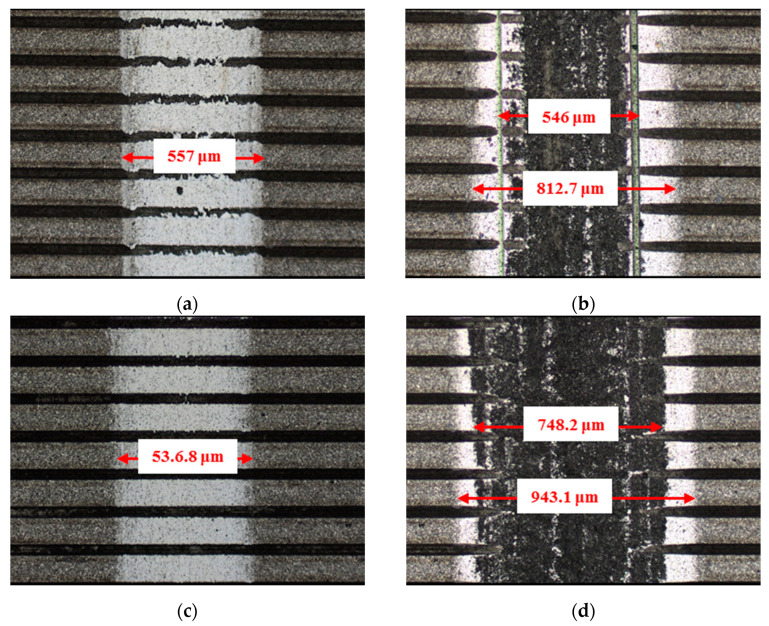

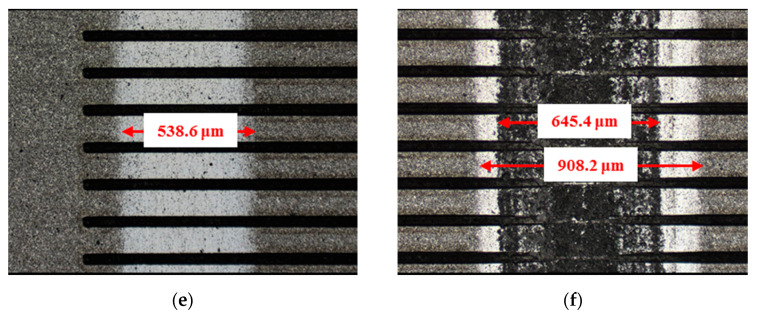
Wear performance of groove-textured SiC under dry-friction conditions with a load of 30 N (×10): (**a**) 3 μm groove-textured SiC–steel ball, (**b**) 3 μm groove-textured SiC–Si_3_N_4_ ball, (**c**) 5 μm groove-textured SiC–steel ball, (**d**) 5 μm groove-textured SiC–Si_3_N_4_ ball, (**e**) 10 μm groove-textured SiC–steel ball, and (**f**) 10 μm groove-textured SiC–Si_3_N_4_ ball.

**Figure 10:**
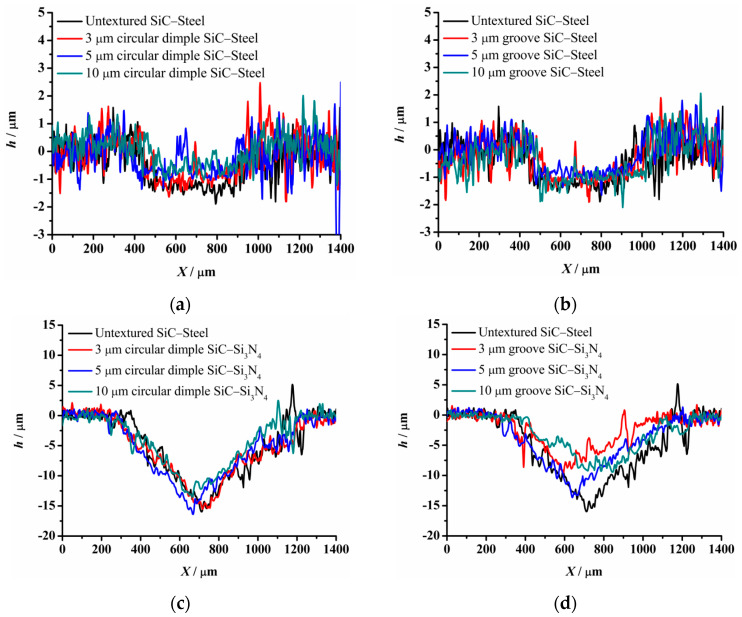
Cross section of wear scars under dry-friction conditions with a load of 30 N: (**a**) circular-dimple-texture SiC–steel ball, (**b**) groove-textured SiC–steel ball, (**c**) circular-dimple-textured SiC–Si3N4 ball, and (**d**) groove-textured SiC–Si3N4 ball.

**Figure 11:**
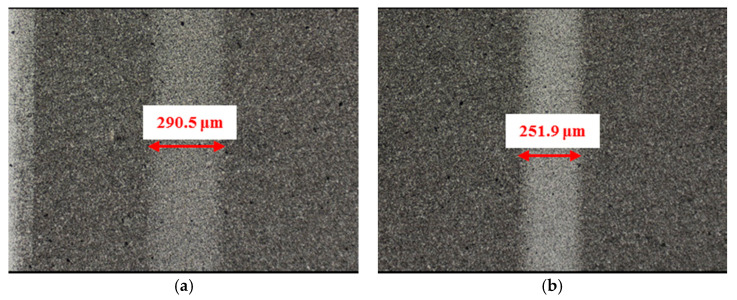
Wear performance of un-textured SiC under oil-lubricated conditions with a load of 75 N (×10): (**a**) untextured SiC–steel ball and (**b**) un-textured SiC–Si_3_N_4_ ball.

**Figure 12:**
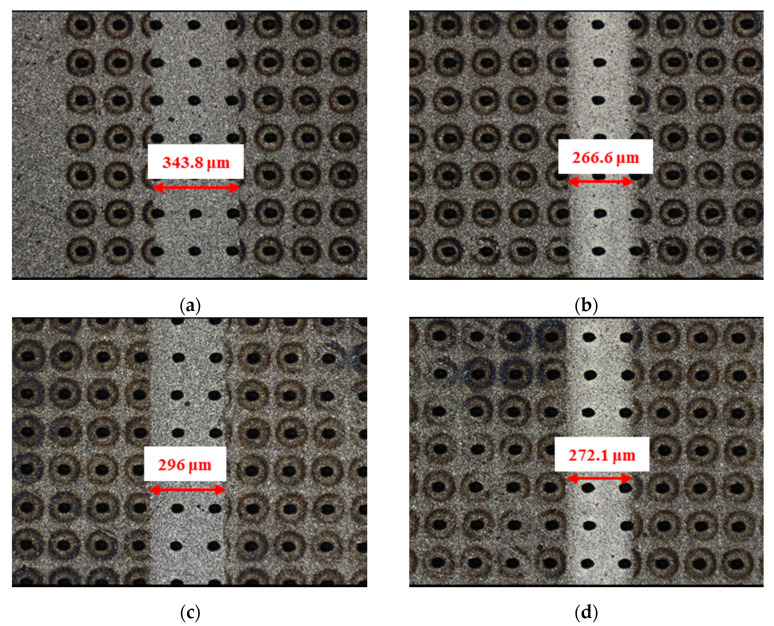

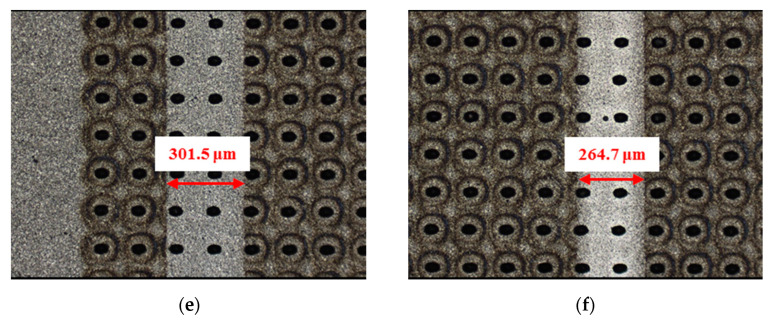
Wear performance of circular-dimple-textured SiC under oil-lubricated conditions with a load of 75 N (×10): (**a**) 3 μm circular-dimple-textured SiC-steel ball, (**b**) 3 μm circular-dimple-textured SiC–Si_3_N_4_ ball, (**c**) 5 μm circular-dimple-textured SiC–steel ball, (**d**) 5 μm circular-dimple-textured SiC–Si_3_N_4_ ball, (**e**) 10 μm circular-dimple-textured SiC–steel ball, and (**f**) 10 μm circular-dimple-textured SiC–Si_3_N_4_ ball.

**Figure 13:**
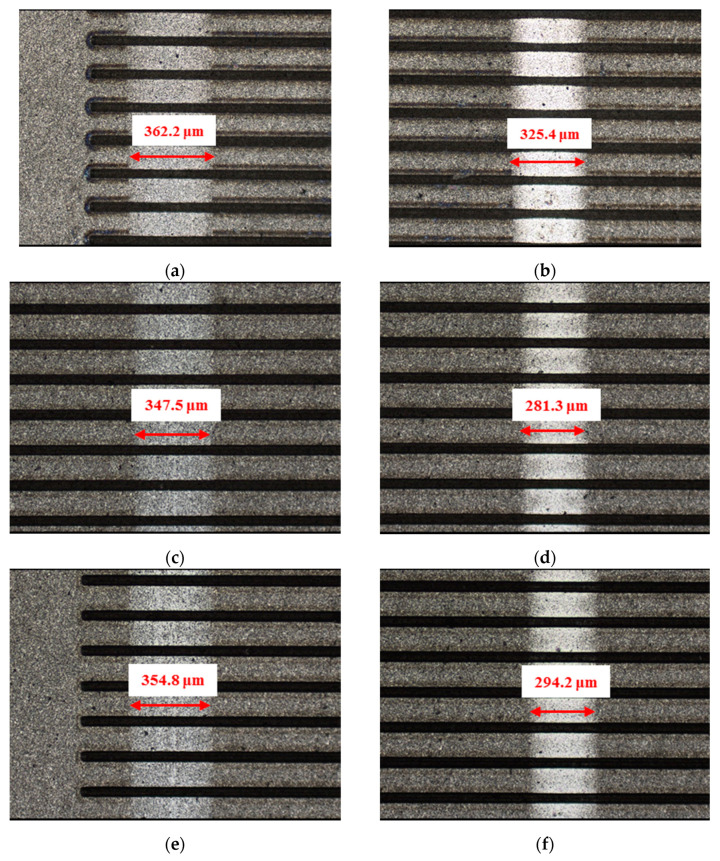
Wear performance of groove-textured SiC under oil-lubricated conditions with a load of 75 N (×10): (**a**) 3 μm groove-textured SiC–steel ball, (**b**) 3 μm groove-textured SiC–Si_3_N_4_ ball, (**c**) 5 μm groove-textured SiC–steel ball, (**d**) 5 μm groove-textured SiC–Si_3_N_4_ ball, (**e**) 10 μm groove-textured SiC–steel ball, and (**f**) 10 μm groove-textured SiC–Si_3_N_4_ ball.

**Figure 14:**
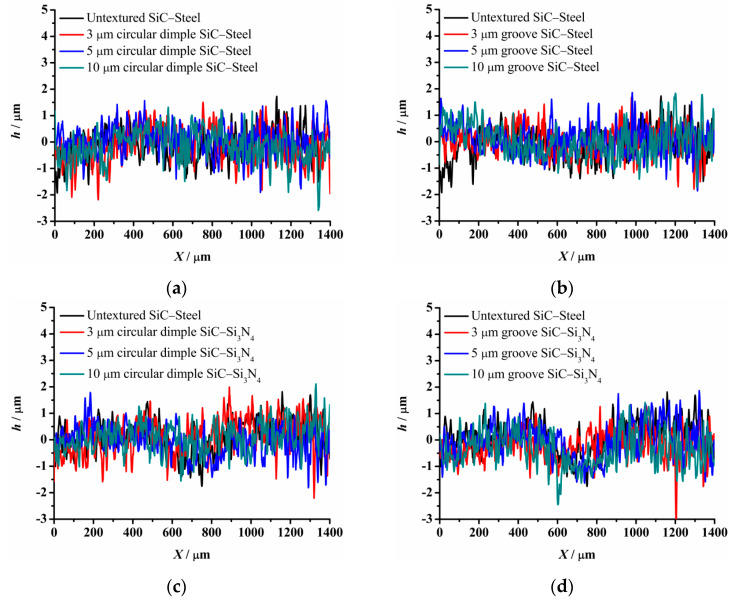
Cross section of wear scars under oil-lubricated conditions with a load of 75 N: (**a**) circular-dimple-textured SiC–steel ball, (**b**) groove-textured SiC–steel ball, (**c**) circular-dimple-textured SiC–Si_3_N_4_ ball, and (**d**) groove-textured SiC–Si_3_N_4_ ball.

**Figure 15:**
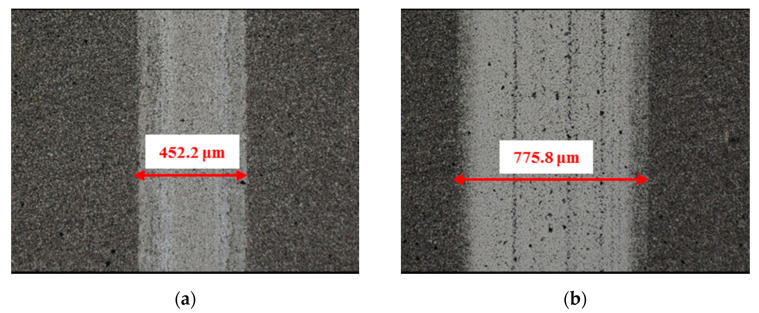
Wear performance of un-textured SiC under water-lubricated conditions with a load of 75 N (×10): (**a**) un-textured SiC–steel ball and (**b**) un-textured SiC–Si_3_N_4_ ball.

**Figure 16:**
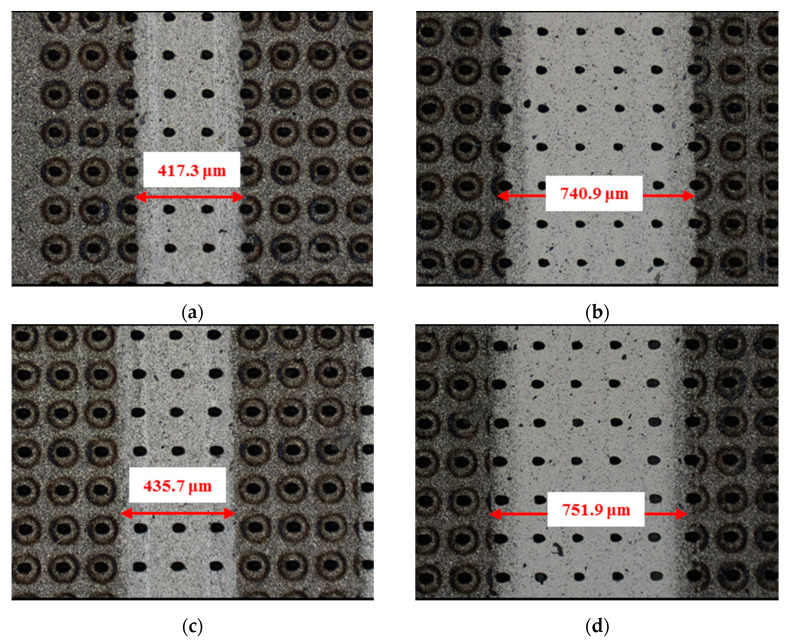

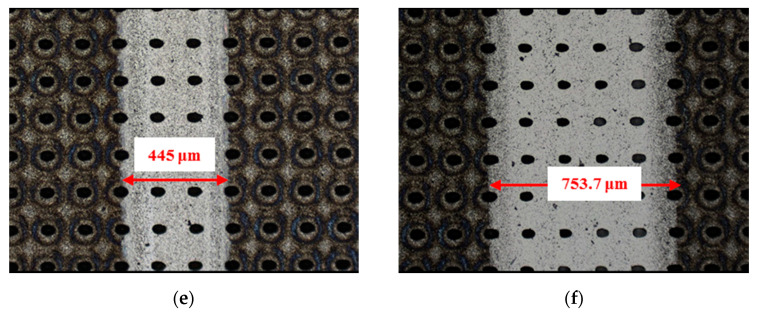
Wear performance of circular-dimple-textured SiC under water-lubricated conditions with a load of 75 N (×10): (**a**) 3 μm circular-dimple-textured SiC–steel ball, (**b**) 3 μm circular-dimple-textured SiC–Si_3_N_4_ ball, (**c**) 5 μm circular-dimple-textured SiC–steel ball, (**d**) 5 μm circular-dimple-textured SiC–Si_3_N_4_ ball, (**e**) 10 μm circular-dimple-textured SiC–steel ball, and (**f**) 10 μm circular-dimple-textured SiC–Si_3_N_4_ ball.

**Figure 17:**
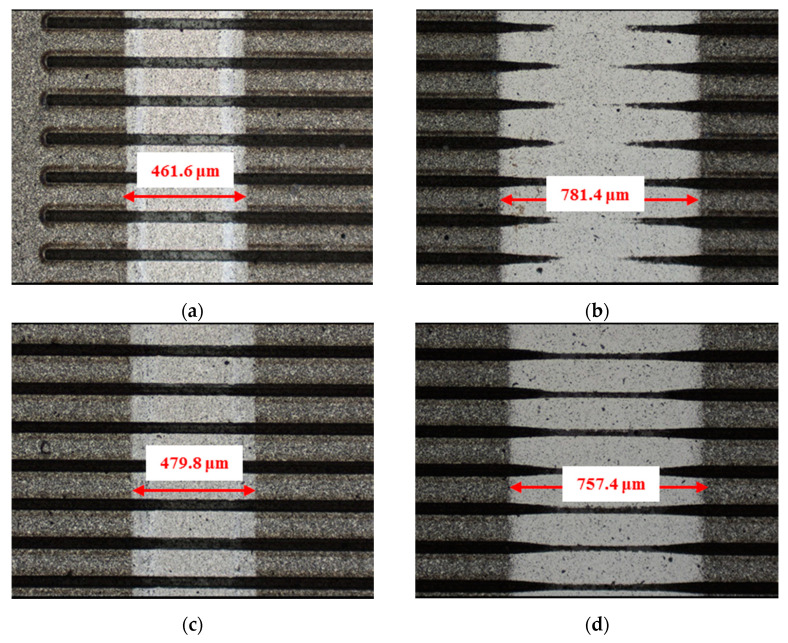

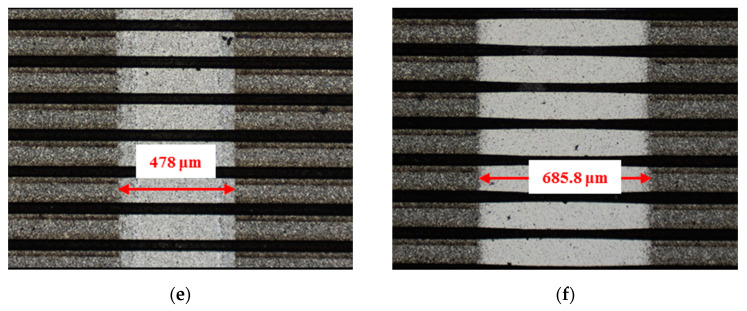
Wear performance of groove-textured SiC under water-lubricated conditions with a load of 75 N (×10): (**a**) 3 μm groove-textured SiC-steel ball, (**b**) 3 μm groove-textured SiC–Si_3_N_4_ ball, (**c**) 5 μm groove-textured SiC–steel ball, (**d**) 5 μm groove-textured SiC–Si_3_N_4_ ball, (**e**) 10 μm groove-textured SiC–steel ball, and (**f**) 10 μm groove-textured SiC–Si_3_N_4_ ball.

**Figure 18:**
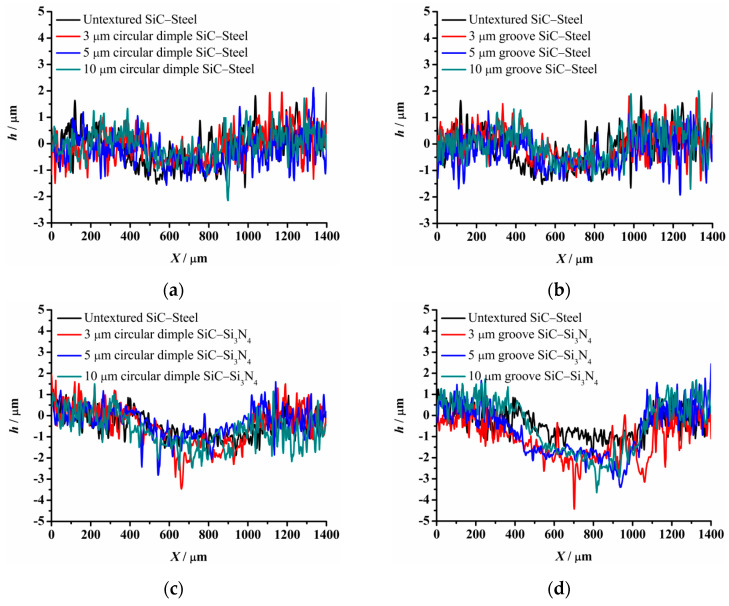
Cross section of wear scars under water-lubricated conditions with a load of 75 N: (**a**) circular-dimple-textured SiC–steel ball, (**b**) groove-textured SiC–steel ball, (**c**) circular-dimple-textured SiC–Si_3_N_4_ ball, and (**d**) groove-textured SiC–Si_3_N_4_ ball.

The material characteristics of friction pairs also affect the friction and wear performance of the textured surface. Experimental results in this research demonstrated that the friction and wear behavior of friction pairs SiC–steel, SiC–Si_3_N_4_, and SiC–PTFE evidence significantly dissimilar trends. Figure 7, Figure 8, Figure 9, Figure 10, Figure 11, Figure 12, Figure 13, Figure 14, Figure 15, Figure 16, Figure 17 and Figure 18 show optical micro-graphs with cross sections of wear scars on the plates when a steel ball and a Si_3_N_4_ ball were used as the interfacing friction materials. The wear scar width of circular-dimple-textured surfaces and groove-textured surfaces was larger than that of the un-textured surface when a steel ball was used, as shown in Figure 7, Figure 8 and Figure 9 under dry-friction conditions. At the same time, the depth of wear scars on the textured surface was similar to that on the un-textured surface, as shown in Figure 10a,b. This shows that though the textured surface can reduce the friction coefficient, it has no effect on reducing wear. It can be seen from Figure 9a that severe adhesive wear occurred on the surface and the coefficient of friction increased. For the Si_3_N_4_ ball-bearing element material, the friction coefficient and the wear scar width were similar to those of the un-textured surface when the depth of the circular-dimple texture was 3 μm and 5 μm. Both the coefficient of friction and the width of the wear scar were lower than those of the un-textured surface when the depth was 10 μm. Therefore, 10 μm circular-dimple textures played an important role in reducing friction and wear. When considering a groove-textured surface, although the friction coefficient showed an insignificant gradient as compared to the un-textured surface, the width of the wear scar was relatively shallow, especially in the middle of the severely worn area. This implies that when the interfacing pair was Si_3_N_4_, the groove textures had a significant effect on wear reduction. The wear depth of the textured surface with various groove depths was smaller than that of the non-textured surface, as shown in Figure 10c,d, which also proved that grooves have excellent friction-reducing performance in groove-textured SiC–Si_3_N_4_ friction pairs.

The wear scars and cross-sectional profiles of the test plates subjected to oil-lubricated conditions are shown in Figure 11, Figure 12, Figure 13 and Figure 14. For Si_3_N_4_ as the bearing material, the wear scar width of both circular-dimple-textured and groove-textured surfaces were larger than those of the un-textured surface. The wear scar width of the circular-dimple texture was smaller than that of the groove texture. At the same time, it can be seen from Figure 14 that the textures had almost no effect on reducing the wear depth for two types of friction pairs. As described before, when steel balls were used, the friction coefficient of the circular-dimple-textured surface was lower than that of the un-textured surface, indicating that the oil supply capacity of the dimple reduced the friction coefficient. However, the groove-textured surface had a higher contact pressure, leading to an increase in the coefficient of friction. For the Si_3_N_4_ ball-bearing element, the contact pressure was relatively higher, and the positive effect of the textures could not have offset the adverse effects, which were caused by the increased pressure; therefore, the friction coefficient and wear were enhanced compared to the non-textured surface.

Figure 15, Figure 16, Figure 17 and Figure 18 show the wear scars and cross-sectional profiles of the plate subjected to water-lubricated conditions. The wear scar width of the circular-dimple-textured surface was smaller than that of the un-textured surface for the steel ball-bearing element material. However, for the groove-textured surface, the wear scar width was larger than that of the un-textured surface. As the wear scar depth of the two textured surfaces was almost the same as that of the un-textured surface, circular dimples with various depths had a good effect on reducing wear. However, groove textures showed no improvement of friction and wear performance under water lubrication conditions. When the interacting friction pair was the Si_3_N_4_ ball-bearing element, the wear scar width of the circular-dimple-textured surface and groove-textured surface with various depths was smaller than that of the un-textured surface, except for 3-μm-deep grooves. For the wear scar depth, the values for circular-dimple-textured surface were almost the same as those for the un-textured surface, but for the groove-textured surface, the values were larger than those for un-textured surface. When the groove depth was 3 μm, the texture wear was more severe compared to other groove textures; therefore, the friction coefficient significantly fluctuated.

As shown in Figure 7, Figure 8, Figure 9, Figure 10, Figure 11, Figure 12, Figure 13, Figure 14 and Figure 15, for steel or Si_3_N_4_ as the ball-bearing element material, there was evidence of wear scar on the surface. However, for PTFE as the interfacial element material, no wear scar was observed after the plate was cleaned. The PTFE ball was worn at the beginning of the test, and the material was transferred to the SiC surface due to the relatively soft PTFE property. Friction and wear therefore occurred between the PTFE ball and the PTFE material transferred to the SiC surface, in turn resulting in a lower friction coefficient. On the textured SiC surface, no wear scar was observed after cleaning, as observed on the un-textured surface when a PTFE ball was used. The PTFE ball was the first to be worn, and then texture filling occurred, leading to severe wear and larger friction coefficient values between the textured surface and the PTFE ball-bearing element.

## 5. Conclusions

The frictional and wear performance of a textured surface against several types of materials under dry, oil-lubricated, and water-lubricated conditions were experimentally studied in this paper. The friction coefficient and wear scar width and depth of un-textured, circular-dimple-textured, and groove-textured surfaces were examined and compared. Considering the experimental results, the following conclusions can be drawn:

1. Texture depth and texture shape affected the friction and wear performance of textured surfaces. However, the influence varied under dry, oil-lubricated, and water-lubricated conditions. This is due to the different effects textures played under various lubricant conditions. The impact of texture was a comprehensive result of the positive role of enhancing the fluid hydrodynamic effect, storing the lubricant, and accommodating abrasive grains and the negative effect of increasing contact pressure.
2. The material of the mated friction pair affected the friction and wear performance of the textured surface. The experimental results in this research showed that the friction and wear behavior of friction pairs SiC–steel, SiC–Si_3_N_4_, and SiC–PTFE show various trends. The circular-dimple texture and the groove texture played a positive role in improving friction and wear performance under certain operating conditions used in this research for SiC–steel and SiC–Si_3_N_4_ friction pairs, but there was absolutely no friction and wear improvement for the textured SiC–PTFE friction pair.

## Figures and Tables

**Table 1:** Experimental conditions.

Load	Stroke	Sliding Frequency	Temperature	Test Time
30 N, 75 N	6 mm	3 Hz	20 °C	300 s

**Table 2:** Sample properties.

Sample	Material	Elastic Modulus	Poisson’s Ratio	Roughness
Plate	SiC	410 GPa	0.16	100 nm
Ball-1	52100 Steel	210 GPa	0.3	20 nm
Ball-2	Si_3_N_4_	320 GPa	0.26	10 nm
Ball-3	PTFE	288 MPa	0.4	150 nm

**Table 3:** Parameters of PAO4.

Lubricant	Viscosity (20 °C)	Density (20 °C)	Viscosity Index
PAO4	35 mPa·s	0.82 g/cm^3^	123

**Table 4:** Test programs.

No.	Lubrication	Load	Lubricant	Plate Texture	Ball
1	Dry	30 N	Air	Circular dimple (3 μm, 5 μm, 10 μm)	Ball-1, Ball-2, Ball-3
2	Dry	30 N	Air	Groove (3 μm, 5 μm, 10 μm)	Ball-1, Ball-2, Ball-3
3	Dry	30 N	air	Smooth	Ball-1, Ball-2, Ball-3
4	Flooded	75 N	PAO4	Circular dimple (3 μm, 5 μm, 10 μm)	Ball-1, Ball-2, Ball-3
5	Flooded	75 N	PAO4	Groove (3 μm, 5 μm, 10 μm)	Ball-1, Ball-2, Ball-3
6	Flooded	75 N	PAO4	Smooth	Ball-1, Ball-2, Ball-3
7	Flooded	75 N	Water	Circular dimple (3 μm, 5 μm, 10 μm)	Ball-1, Ball-2, Ball-3
8	Flooded	75 N	Water	Groove (3 μm, 5 μm, 10 μm)	Ball-1, Ball-2, Ball-3
9	Flooded	75 N	Water	Smooth	Ball-1, Ball-2, Ball-3

**Table 5:** Contact conditions.

Lubrication	Load	Ball	Contact Pressure	Contact Radius
Dry	30 N	Ball-1 (steel)	1.73 GPa	91 μm
		Ball-2 (Si_3_N_4_)	2.02 GPa	84 μm
		Ball-3 (PTFE)	0.03 GPa	690 μm
Flooded	75 N	Ball-1 (steel)	2.34 GPa	124 μm
		Ball-2 (Si_3_N_4_)	2.75 GPa	114 μm
		Ball-3 (PTFE)	0.04 GPa	936 μm

## Data Availability

The data are not publicly available due to the work is ongoing.
